# Iatrogenic Cardiac Tamponade Secondary to Central Venous Catheter Placement: A Literature Review

**DOI:** 10.7759/cureus.37695

**Published:** 2023-04-17

**Authors:** Angelo A Messina Alvarez, Mohammad A Bilal, Nouraldeen Manasrah, Ahmed Chaudhary

**Affiliations:** 1 Internal Medicine, Detroit Medical Center (DMC) Sinai-Grace Hospital, Detroit, USA

**Keywords:** mortality, incidence, myocardial perforation, pericardial effusion, central venous catheter, cardiac tamponade

## Abstract

Cardiac tamponade is the fluid accumulation within the pericardial sac that compresses the heart and decreases cardiac output. More than 20% of the cases are surgical or non-surgical iatrogenic causes. Cardiac tamponade has been described as a rare complication of central venous catheter placement with an incidence in adults as low as less than 1% but with significantly high mortality of more than 60%. The purpose of this article is to review the incidence, clinical manifestations, pathophysiology, diagnosis, and management of cardiac tamponade after central venous catheter placement as well as different methods to prevent this fatal complication from occurring.

## Introduction and background

Cardiac tamponade is a medical emergency characterized by fluid accumulation within the pericardial sac, compressing the heart and decreasing cardiac output, leading to cardiogenic shock [1]. Approximately 21% of cardiac tamponade cases are iatrogenic due to surgical and non-surgical interventions. Among the non-surgical reasons, fibrinolytic therapy for acute myocardial infarction, anticoagulation, or anticancer therapy is included. Meanwhile, percutaneous coronary intervention, catheter ablation, percutaneous valvuloplasty, or pacemaker implantation represents procedural causes of iatrogenic cardiac tamponade [2].

Central venous catheters are the most common invasive procedure in critical illness treatment that is crucial for the management of most patients who are in the intensive care unit as it can be used for the administration of solutions with high osmolarity, parenteral nutrition, blood transfusion, vasopressors, hemodynamic monitoring, or renal replacement therapy [3].

Previously, placement was mainly guided by anatomical features and position. However, ultrasound-guided line insertion is currently the preferred method to decrease the number of complications from this procedure. Still, approximately 20% of insertions are associated with pneumothorax, hemothorax, and thrombosis, among others [3].

Nevertheless, cardiac tamponade is an uncommon but fatal complication of centrally inserted venous catheters. This review aims to evaluate and summarize information regarding the characteristics of this complication and provide multiple recommendations to prevent it from happening and management options [3].

## Review

Anatomy

The pericardium consists of two layers and a virtual space: The outer layer is called parietal pericardium, and the inner layer is called the visceral pericardium; within both covers is the pericardial space that is filled by approximately 15-50 ml of pericardial fluid, which is a plasma ultrafiltrate that allows proper motion between the layers of the pericardium [4].

Pathophysiology

Cardiac tamponade develops as the accumulation of fluid within the pericardial space, which can impair the diastolic filling and ultimately decrease the stroke volume and therefore cardiac output [5].

This process can be slow and progressive, where patients can accumulate a significant amount of fluid within the space over time, but the slow progression allows the stretching of the pericardium. On the other hand, a small but rapid accumulation of fluid would not give time for the parietal pericardium to expand, causing restriction in cardiac filling and provoking acute and significant hemodynamic instability [5].

For example, adults can tolerate a chronic buildup of pericardial fluid of up to 1500 ml, but a rapid accumulation of 100-350 ml can be deadly. In pediatric patients, given their smaller dimensions, an accumulation of only 50 ml can be mortal [6].

The mechanism of how central venous catheter insertion causes iatrogenic cardiac tamponade is based on the cardiac wall perforation, which can be either immediate or delayed.

Immediate

Puncture of the vena cava or the heart is the least common as it is usually a consequence of using stiff and sharp catheters [7].

Delayed

The catheter tip is in direct contact with the endocardium, which damages the wall during each contraction and creates a thrombus. Eventually, the catheter tip induces necrosis of the area where it is making contact and then finally perforates the inner layer of the heart. This process is even faster when the patient has infiltrative heart conditions or heart failure [7].

Another cause of delayed cardiac tamponade is the administration of high cytotoxic or hypertonic and high-pressure infusions through central venous catheters that can develop cardiac tamponade over the course of days to months [8].

Incidence

The incidence of cardiac tamponade secondary to central venous catheters is challenging to determine, primarily due to the high mortality of this condition. In adults, it is between 0.0001% and 1.4%. In pediatric patients requiring central venous catheter placement, the incidence of cardiac tamponade is higher, in 1%-3% of cases [9].

Bar-Joseph et al. reported that the increased incidence of cardiac tamponade in pediatric patients is related to their smaller dimension often and catheter length, which increases the risk of the catheter tip being inside the cardiac cavities and being in contact with the endocardial wall, therefore increasing the risk of perforation [10].

A study that took the information from the American Society of Anesthesiology Closed Claims Project database reviewed more than 6400 claims associated with adverse outcomes from medical practice between 1970 and 2004. Around 110 complications were related to central venous catheters, and 16 were cases of cardiac tamponade, representing 14.5% [11].

Risk factors

Multiple operator-dependent and non-operator-dependent elements increase the possibilities of causing iatrogenic cardiac tamponade (Table 1).

**Table 1:** Risk factors for iatrogenic cardiac tamponade after central venous catheter placement

Risk factors	Association of risk factors with iatrogenic cardiac tamponade
Insertion site	Cardiac tamponade is more common when the catheter is placed peripherally (cephalic, basilic, or brachial vein) compared to centrally placed (internal jugular vein or subclavian vein). This is because the abduction or extension of the arm can move the catheter tip by 7 cm in comparison to movements of the head and neck where the catheter tip can move only by 2 cm due to the restricted movements of those areas [12].
Skin fixation and suturing of the catheter	Improper adhesion and suturing of the catheter to the skin can lead to an advanced movement of the tip of up to 10 cm [12].
Angle and positioning	There is an increased risk of damage to the endocardial wall when the catheter tip is placed at a 90-degree angle to the wall [12].
Guidewire type	There is an increased risk of perforation when using a non-curved metallic guidewire in comparison to a J-tip guidewire, given the fact that it can easily puncture the myocardium causing rupture and erosion [12].
Catheter material	Flexible catheters such as “pig-tail catheter” has shown a decreased probability of endocardial wall rupture in comparison to rigid catheters [12].

Common areas of injury

A study performed by the Departments of Cardiology and Cardiothoracic Surgery at the Dupuytren Hospital in Limoges, France, in 1988 summarized 50 published cases of iatrogenic cardiac tamponade after central venous catheters and detailed the areas most affected, which were right atrium in 29 cases that represented 43%, right ventricle in 18 patients (27%), and finally, the superior vena cava in three cases being 4% of the total [13].

A retrospective study performed by Collier looked for the mechanism of how patients developed cardiac tamponade after central venous catheter insertion, which included 25 unreported cases of this complication in which the catheter insertion site was determined in 80% of the cases: 15 occurred in the right atrium (60%), four in the right ventricle (16%), and one in the pericardial part of the cava-atrial junction (4%) [14].

Clinical presentation

Signs and symptoms can vary in time depending on the severity of the injury. According to a study performed in the Department of General Surgery at the Felicio Rocho Hospital in Belo Horizonte, Brazil, sudden death presents without any warning symptomatology in 29% of cases [15].

Most patients can present signs and symptoms during the first week, and approximately 36% of the cases can show clinical features in the first 24 hours [15].

The most common symptoms are pain or discomfort in the chest or epigastric area, nausea, and dyspnea, while a physical exam may reveal tachycardia, elevated jugular venous pressure, hypotension [15], and paradoxical pulse, which is defined as an inspiratory decrease of systolic blood pressure by more than 10 mmHg [16].

Diagnosis

The diagnosis of cardiac tamponade is clinical, based on the combination of symptoms known as Beck’s triad (elevated jugular venous pressure, hypotension, and muffled heart sounds). However, in cases of acute cardiac tamponade after central venous catheter placement, these symptoms may not be present in up to 29% of the cases, and the patient can die from vague symptoms [9,17].

On EKG, patients can show low cardiac voltages and signs of electrical alternans, and chest x-ray can show an enlarged cardiac silhouette [10]. However, any patients presenting the above-mentioned symptoms may not have EKG changes or enough fluid accumulation to be seen in chest x-rays [9].

The main diagnostic test to confirm the presence of cardiac tamponade is an echocardiogram, which is preferred to be performed acutely due to its availability and the fact that few echocardiographic findings suggestive of tamponade can precede the clinical signs and aid with early detection of this complication. It has a sensitivity ranging from 90% to 100% and a specificity of 70%-100% [18].

**Figure 1:**
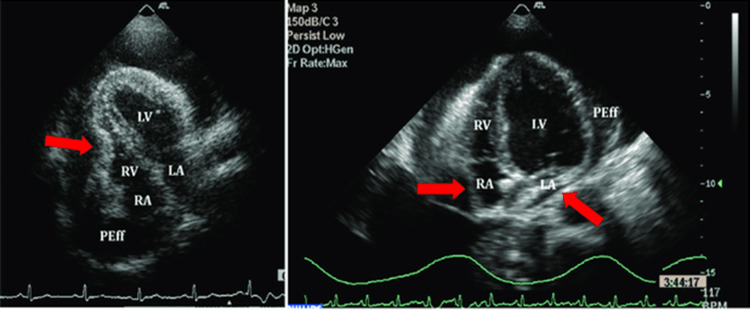
Right ventricular collapse on echocardiogram Source: Ref. [19].

**Figure 2:**
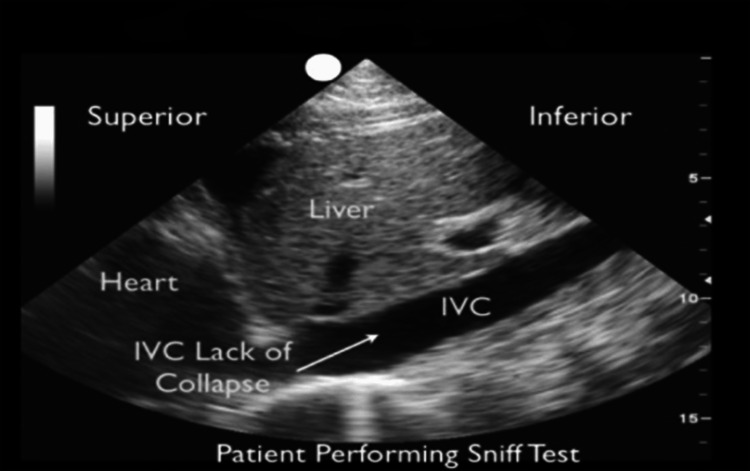
Dilated IVC and lack of IVC collapse IVC: Inferior vena cava. Source: Ref. [20].

Features observed in 2D-echocardiogram that can guide diagnosis of cardiac tamponade are right atrial and right ventricle collapse (Figure 1) during diastole, dilated inferior vena cava, lack of inferior vena cava collapse during inspiration (Figure 2), swinging heart, and significant respiratory variation in cardiac filling observed by assessment of trans-mitral and trans-tricuspid flows [5].

Management

When cardiac tamponade is suspected or confirmed, the first step is to stop the infusion of any fluid or medication going through the central venous catheter. The next step is to lower the perfusion container below the level of the heart and aspirate. This generally empties the pericardial sac and allows recovery of the cardiac output [7].

If symptoms and vital signs do not improve, the next step is to remove the central venous catheter and perform pericardiocentesis [21].

Pericardiocentesis can either be performed without imaging assistance by positioning the patient in the Trendelenburg position or by using an 18-20-gauge needle to puncture just below the xiphoid/left costal junction in a 45-degree angle in the direction of the left shoulder. This process is performed with constant aspiration until there is a return of fluid. Alternatively, there is a right parasternal approach at the level of the third and fourth intercostal space [22]. Currently, ultrasound-guided pericardiocentesis is preferred as it can be performed with a more accurate anatomical approach, thereby lowering the risk of complications from this procedure [22].

Finally, if, despite pericardiocentesis, hypotension remains severe and vital signs continue to deteriorate, a thoracotomy should be performed [21].

Mortality

The mortality rate in adults that develop cardiac tamponade created by perforation from central venous catheter placement is 65%-100%. On the other side, in children, despite having a higher incidence of developing cardiac tamponade, the mortality rate is lower, between 30% and 50% of the cases [4]. For example, Karnauchow reviewed 59 cases described in the literature between 1968 and 1984, with a mortality rate of 69% [23]. Meanwhile, Iglesias et al. from the Cardiovascular Surgery Department at Clinica Puerta Hierro in Madrid, Spain, found that from 21 cases of cardiac tamponade, 19 were fatal, representing a mortality rate of 90% [24].

Finally, Greenall listed a total of 16 cases of cardiac tamponade after central venous catheter described in the literature from 1968 to 1975, of which 14 were fatal, and 50% of the deaths were observed in the first 24 hours post-insertion [25].

Prevention

As mentioned before, iatrogenic cardiac tamponade is an uncommon but exceptionally dangerous complication of central venous catheters. Ultimately, the prevention of such a problem will depend on the provider’s expertise in performing the procedure, but there are some technical specifications that can reduce the risk (Table 2).

**Table 2:** Recommendations for the prevention of iatrogenic cardiac tamponade secondary to central venous catheter placement

Recommendation	Description
Insertion approach and site confirmation	Catheter position should be checked via x-ray as soon as it is placed and should follow the “Greenall criterion” where the tip should not lie more than 2 cm below an imaginary line that crosses the lower surfaces of the clavicular heads [12,21]. In the case of internal jugular vein approach, the preferred catheter insertion site is as high as possible, while for subclavian approach, it is as lateral as possible [12].
Catheter stability	Catheters should have distal ends properly sutured or secured, especially peripherally inserted central venous catheters [7].
Catheter check	Frequent checks of the catheter for the absence of backflow or abnormally high central venous pressure [7] should be done.
Guidewire placement technique	Avoid inserting the guidewire for a length of more than 18 cm. In addition, avoid pushing the guidewire when resistance is noted, instead pullback and reinsert [26].
Point-of-care ultrasound	Point-of-care ultrasound-guided central venous catheter insertion has proved to decrease the risk of cardiac complications [9,11].

Positioning is a key factor to prevent cardiac tamponade as direct contact with the endocardial wall can lead to perforation; therefore, the catheter tip should not be placed either in the right ventricle or right atrium. Fletcher and Bodenham described that the best place for tip positioning is the superior vena cava, specifically the lower vena cava near the entrance to the right atrium or the upper vena cava. The preferred method to confirm proper catheter positioning and no tip coiling is a chest x-ray [27].

On the other hand, catheter characteristics per se can represent a risk factor for developing cardiac tamponade while performing this procedure. However, there are specific catheter characteristics that help reduce the risk of cardiac perforation while placing a central venous catheter (Table 3).

**Table 3:** Catheter characteristics that reduce the incidence of iatrogenic cardiac tamponade secondary to central venous catheter placement CVC: Central venous catheter.

Equipment characteristics and infusion parameters	Description
Catheter material	Polyethylene or nylon catheters are not used as they are rigid. Silicone catheters (Silastic) are preferred [22].
Rate of infusion through catheters	Central venous catheters should not be used to rapidly deliver isotonic solutions when other peripheral routes are available as this increases the risk of injury to the endocardial wall [7].
Guidewire type	J-tip guidewires should be used, and using sharp guidewires should be avoided [26].
Catheter length	For adults, the use of short-length catheters is preferred (15-16 cm), which is proven to prevent intracardiac catheter placement [9]. A study conducted at the Texas Children’s Hospital on 330 pediatric patients who had CVC placement established a formula for the proper length of insertion: If the patient’s height is less than 100 cm, the formula is (height in cm/10)-1; and if the patient height is more than 100 cm, the formula is (height in cm/10)-2 [28].

## Conclusions

Cardiac tamponade is one of the rarest and most dangerous complications of central venous catheter placement. Most of the patients present with the classic Beck triad. However, many patients can die by showing only vague symptoms. It has a very high mortality rate, especially when the fluid accumulates fast within the pericardial sac. Multiple risk factors are associated with this complication and can be either related to the provider placing the catheter or the catheter characteristics or materials per se.

The diagnosis is primarily clinical, and an echocardiogram can confirm the presence of pericardial effusion; however, prompt management is required, primarily with discontinuation of IV fluids and ultrasound-guided pericardiocentesis as key components in the treatment of cardiac tamponade. If these treatment options fail, thoracotomy can be performed as well. Using silicone catheters, J-tip guidewires, not advancing the catheter further than recommended, proper catheter securing, and imaging confirmation of placement are vital methods to prevent cardiac tamponade from central venous catheter placement.
